# Study on the Constitutive Relationship between Ordinary Concrete and Nano-Titanium Dioxide-Modified Concrete at High Temperature

**DOI:** 10.3390/ma16144910

**Published:** 2023-07-09

**Authors:** Dongpeng Wu, Zhicheng Wang, Yungui Pan, Jian Huang, Tomás Manuel Fernández-Steeger, Chao Xu, Xinlong Tang, Zhiyu Long, Yufei Tang

**Affiliations:** 1 Department of Civil Engineering, School of Environment and Architecture, University of Shanghai for Science and Technology, Shanghai 200093, China; 2 Institut Für Angewandte Geowissenschaften, Technische Universität Berlin, Ernst-Reuter-Platz 1, BH 3-1, 10587 Berlin, Germany

**Keywords:** concrete, nano SiO_2_, high temperature, stress–strain relationship, damage model

## Abstract

After high-temperature treatment, both nano-titanium dioxide-modified concrete and ordinary concrete exhibit typical splitting failure. High-temperature heating reduces the mechanical properties and brittleness of concrete and improves the ductility of concrete. The stress–strain relationship of the specimens was obtained through the uniaxial compression test of ordinary concrete and nano-titanium dioxide-modified concrete cube specimens under normal temperature and high-temperature conditions. In addition, the relationship between temperature and damage variables was established, and the unified constitutive model containing damage variables after room temperature and high-temperature treatment of ordinary concrete and nano-titanium dioxide-modified concrete were established. It provides a reference for future research on the mechanical properties of high-performance concrete structures after high temperatures (fire).

## 1. Introduction

Under normal room temperature conditions, the general building structure can maintain its intended functionality for an extended period. However, in instances where there exists a substantial temperature differential within the building environment, the structural load-bearing capacity may diminish, leading to a decline in performance and ultimately resulting in structural failure. The incidence of solar radiation on the sun-facing facade during summer months increases the temperature of the exterior surface of the building. However, this temperature generally does not exceed 60 °C, and it will also cause cracks on the surface of the building, affecting the normal use of the building. In addition, some workshops are in a high-temperature environment for a long time, such as some chemical and metallurgical enterprises’ high-temperature workshops, and their structural surface temperature can reach 200 °C or higher. When the chimney emits high-temperature gas, the internal temperature can reach 500~600 °C. In some accidental cases, there will be a short-term high-temperature impact on the building, such as the temperature of the building reaching 1000 °C or higher within one hour during a fire. At high temperatures, the pore pressure and porosity of concrete will change, and phenomena such as thermal expansion, thermal cracking, and thermal creep will occur. These phenomena will destroy the mesoscopic structure of concrete, leading to a gradual decline in its mechanical performance and problems such as cracking and spalling on the surface. In addition, concrete can also cause damage due to bending or stiffness and mass loss, etc. Various studies indicate that the main effect of fire (high temperature) on concrete is a change in its properties, leading to spalling, while the stress–strain behavior under high-temperature exposure is complex.

In recent years, the research on high-temperature damage of concrete at home and abroad has mainly focused on the change law of mechanical properties of concrete under high temperatures [1,2,3,4,5,6,7] and the establishment of a series of high-temperature damage models [8,9,10,11,12,13]. In addition, there are few systematic studies on the damage constitutive relationship of nano-titanium dioxide-modified concrete under high temperatures. Gao et al. [14] prepared the specimens using nano silica and calcium carbonate. Each group was treated at a temperature of 25~800 °C, and the influence of nanoparticles on the compressive characteristics of concrete at high temperatures was studied through uniaxial compression tests. The results showed that the peak stress of nano-silica modified concrete with a content of 1.5% increased by 29.4%, 24%, and 38.7% at room temperature, 400 °C and 800 °C compared with ordinary concrete, respectively. Fu et al. [15] studied the effects of nanomaterials on the residual compressive strength and residual flexural strength of mortar after high-temperature treatment, and the research results showed that after curing at 800 °C, the residual stress of 1.5% nano-silica modified mortar was higher than that of nano-silica modified mortar mixed with 3%. Elkady et al. [16] studied the effect of heating on the mechanical properties of nano-silica concrete. Amounts of 1.5%, 3%, and 4.5% nano-silica were added to the concrete mixture, and after 28 days of natural curing, it was treated at a high temperature of 200~600 °C. After high-temperature treatment at 600 °C, the strength loss of concrete incorporated with 1.5% was the lowest, and the residual compressive strength and bending strength were 73% and 35%, respectively. In comparison to the control mixture that lacked nano-silica, the compressive strength and bending strength exhibited a significant increase of 43% and 38.5%, respectively.

Composite materials along with nanoconcrete have been the subject of extensive research by numerous scholars, with a focus on their mechanical properties [17]. Rawat et al. [18] present a comprehensive review of prior investigations concerning the impact of nano-titanium dioxide on various characteristics of plain or blended cement systems, including workability, setting time, mechanical strengths, water absorption, and porosity. Alobaidi et al. [19] discussed the effects of nano-fly ash particles on the room temperature and high-temperature properties of self-compacting concrete. Under the same displacement rate, the size, shape and consistency of nano fly ash particles and fly ash particles were characterized by scanning electron microscopy, and the differences between nano fly ash particles and fly ash particles were compared. Zhang [20] experimentally studied the splitting tensile, compressive, and flexural strength of ordinary concrete and basalt fiber concrete doped with nano-silica at different temperatures. The results show that the compressive strength, splitting tensile strength and flexural strength of basalt fiber concrete doped with nano silica are higher than those of ordinary concrete at various temperatures. Nikbin et al. [21] cured the heavy concrete containing magnetite aggregate under different temperatures (25, 200, 400, and 600 °C), and replaced part of the cement with 0%, 2%, 4%, and 6% nano-titanium dioxide particles by cement weight, and carried out compressive strength, γ ray shielding test, and scanning electron microscopy analysis of the concrete specimen. Evaluation parameters for radiative attenuation tests include linear attenuation coefficient, half-value layer, tenth value layer, and mean free path. The results show that the ultrasonic wave velocity and compressive strength of the specimen mixed with nano-titanium dioxide first increase and then decrease with the increase in temperature. Bastami et al. [22] studied the effects of compressive strength, tensile strength, spalling, and mass loss of nano-silica modified high-strength concrete at high temperatures. The mechanical properties of the modified high-strength concrete were tested by heating the concrete specimen to four temperatures of 400 °C, 600 °C, and 800 °C at a rate of 20 °C/min. The results show that nano-silica is effectively used in high-strength concrete and can improve its high-temperature mechanical properties. The presence of nano-silica improves the residual compressive strength and tensile strength of the material, and with the increase of permeability, the peeling and mass loss of the material decrease.

This study investigates the effects of various high temperatures on both ordinary concrete and concrete modified with nano-titanium dioxide. Subsequently, an investigation was conducted on the compressive strength of the two distinct categories of concrete subsequent to exposure to elevated temperatures. The damage mechanics approach was utilized to establish the constitutive model for ordinary concrete. In this study, a constitutive model was developed for nano-titanium dioxide-modified concrete to assess its damage. The model utilized the peak strengthening coefficient, and the test data presented in this paper were subjected to comparative and analytical scrutiny. The aforementioned offers a precise point of citation pertaining to the efficacy of concrete constructions subsequent to exposure to elevated temperatures, specifically those resulting from conflagrations.

## 2. Experimental Materials and Equipment

### 2.1. Materials

This study used PO42.5 (Conch Cement Company, Wuhu, China) grade ordinary silicate cement with a relative density of 3.12 g/cm^3^ with good stability. The chemical composition of the cement is listed in Table 1. In addition, limestone-graded gravel, river sand, nano titanium dioxide, water-reducing agent, and tap water were used.

**Figure 1:**
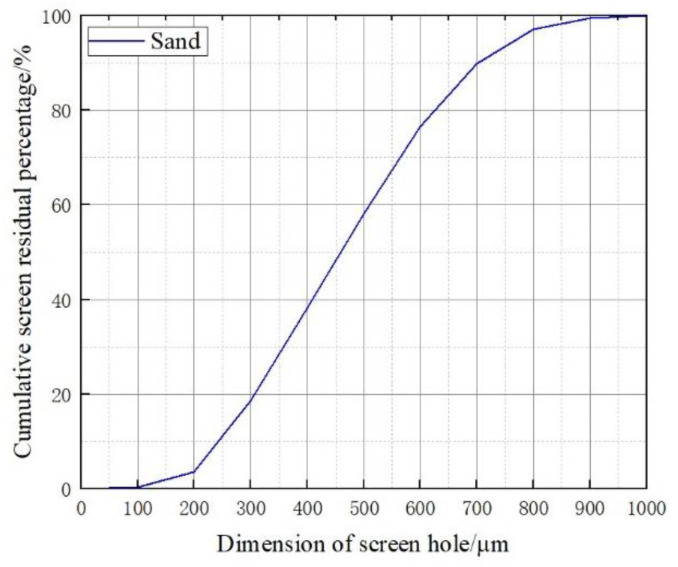
Dimension of screened sand.

Natural and clean river sand was selected and screened with a standard sieve with a pore size of 0.6 mm. The maximum particle size of the test sand is less than 1.2 mm, and most of the particles (more than 99%) are below 900 μm. More than 50% of particles have a particle size of less than 500 μm. The average particle size is 0.46 mm. The gradation curve of river sand is shown in Figure 1.

**Figure 2:**
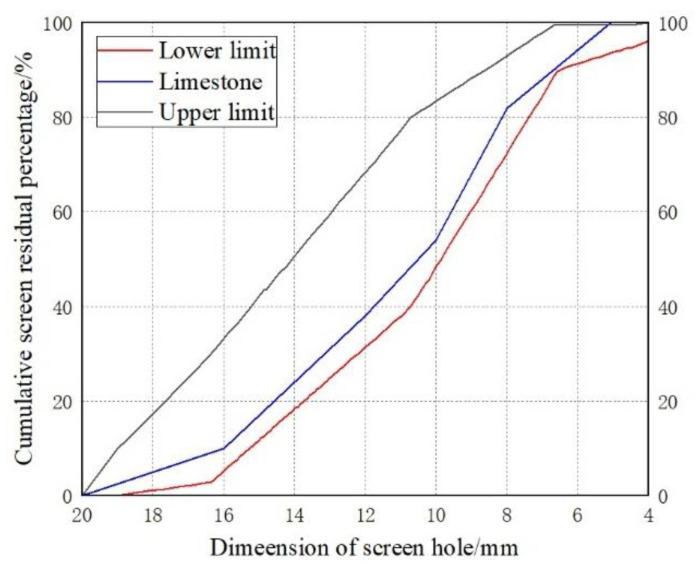
Grading curve of coarse aggregate.

Limestone crushed stone was used, and the particle size is 5–20 mm continuous grading. The crushed stone used for the crushing index test was passed through a 14 mm test sieve and left on a 10 mm sieve. Put the crushed stone on the sieve into a 100–110 °C oven to dry for four hours, then put it into a cylindrical mold to vibrate and compact, and then put the plunger on top of the aggregate. The integral unit was placed on the pressure testing machine, and the plunger was loaded and gradually pressurized to 400 kN within 10 min. After unloading, the crushed stone was taken out and placed in 2.36 mm for screening, and the ratio of the mass of the crushed stone through this screening to the total mass is the crushing index. The crushing index of the coarse aggregate crushed stone used in the test is 4.8%, and the density is 2.7 g/cm^3^. The grading curve of limestone crushed stone is shown in Figure 2.

The water-reducing agent adopted was 2651F (BASF, Heidelberg, Germany), which is a modified polycarboxylic acid ether made by a spray drying process, which has good fluidity and uniform color.

**Figure 3:**
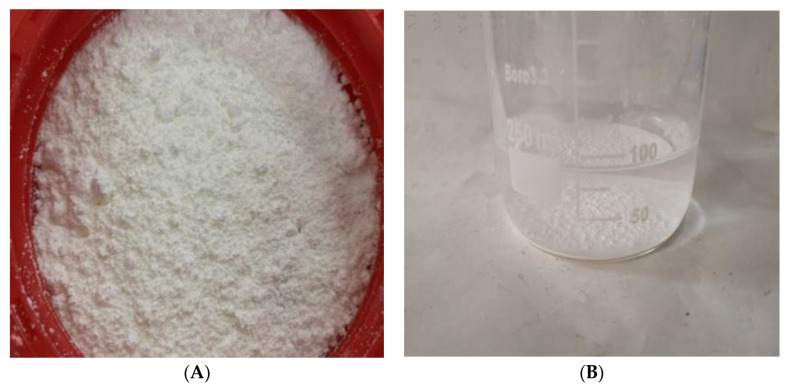
Nano-titanium dioxide. (**A**) nano-titanium dioxide powder. (**B**) aqueous solution of nano-titanium dioxide.

Nano titanium dioxide (Xfnano, Nanjing, China) is in the form of white powder, as shown in Figure 3, and its physical properties are shown in Table 2. When the aqueous solution was prepared, nano titanium dioxide was found to precipitate in the solution, as shown in Figure 3. The even spreading of nano titanium dioxide in concrete can be achieved by utilizing an appropriate amount of water reducing agent and mechanical mixing, as nano titanium dioxide has been shown to be insoluble in water.

### 2.2. Sample Preparation

The mix ratio is listed in Table 3. According to the results of the previous research, the nano titanium dioxide content of 3% (NTC3 and the notation “NTC3” denotes the incorporation of nano-titanium dioxide into cement at a proportion of 3% of the cement mass) was chosen in this paper [23]. The specific preparation and configuration process of nano-titanium dioxide-modified concrete specimens was referred to the research of Xu et al. [24]. Ordinary concrete was prepared the same way as nano-titanium dioxide-modified concrete without adding titanium dioxide. Concrete samples had cubic specimens with 100 mm inside. The test specimens were created and put through testing in accordance with Standard for Testing Methods for Mechanical Properties of Ordinary Concrete (GB 50081-2002) [25]. The curing period for both types of concrete is 28 days.

### 2.3. Test Equipment

**Figure 4:**
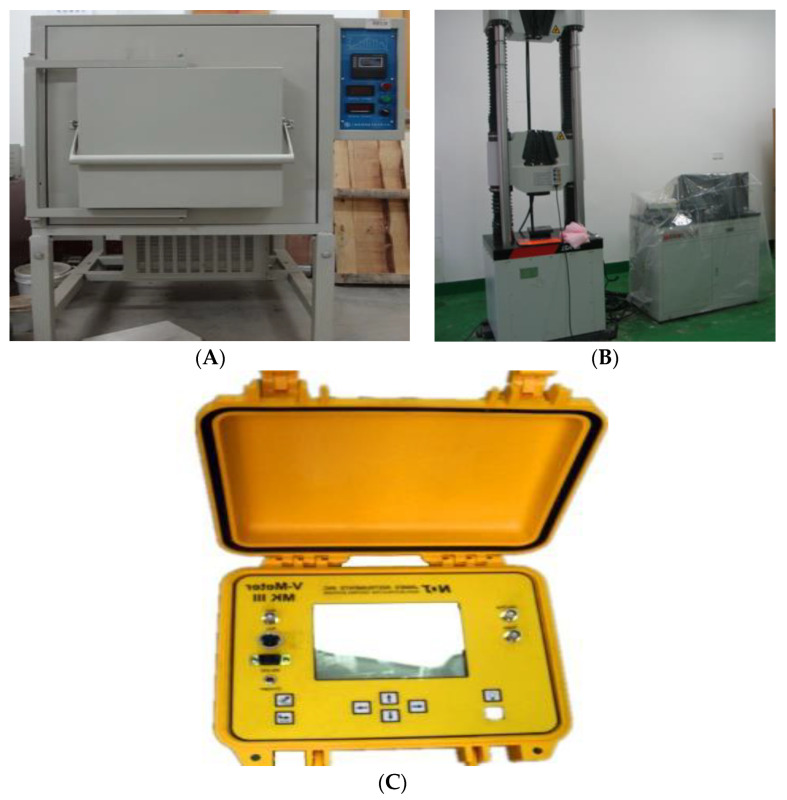
Test equipment. (**A**) SMF1900-50 Chamber Resistance Furnace. (**B**) Rigid Servo Triaxial Compression Tester. (**C**) 2V-METER III Ultrasonic Pulse Velocity Tester.

The instrument for the high-temperature treatment of the specimen is a chamber resistance furnace with model SMF1900-50 (Shanghai Haoyue Vacuum Equipment Co., Ltd., Shanghai, China, Figure 4A), and the maximum heating temperature can reach 1900 °C. The test sample heating process is as follows:

(1) Fifteen ordinary concrete and fifteen NTC3 specimens were carefully chosen and subsequently partitioned into five distinct groups.
(2) After 28 days of natural conservation, it was subjected to high-temperature heat treatment of 200 °C, 400 °C, 600 °C, and 800 °C, respectively. The heating rate in the high-temperature furnace is 5 °C/min. The temperature is constant for 6 h after reaching the target temperature, and then naturally cooled in the furnace for 24 h.

The uniaxial compression testing instrument for concrete specimens is the microcomputer-controlled rigid servo triaxial pressure testing machine produced by Xi’an Lichuang Material Testing Technology Co., Ltd. (Xi’an, China). The maximum load is 2000 kN and the axial load accuracy is ±1%, as shown in Figure 4B. The rate of uniaxial compression test is 0.5 MPa/s.

The longitudinal wave velocity test adopts V-METER III ultrasonic pulse velocity tester (James Instruments Inc., Chicago, IL, USA), as shown in Figure 4C, and the test accuracy is 0.1 μs.

## 3. Test Results and Discussion

### 3.1. Surface Characteristics of Specimens after High Temperature

**Figure 5:**
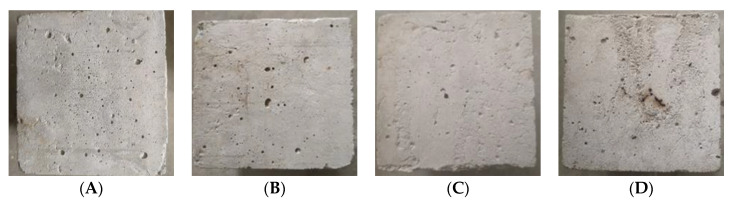

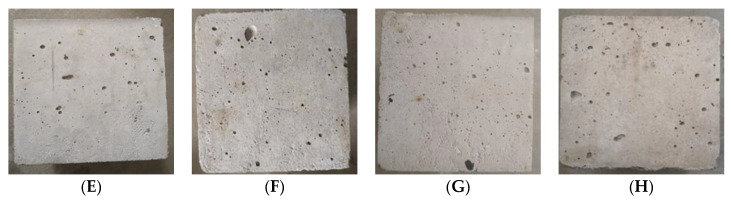
Surface characteristics of ordinary concrete and NTC3 specimens after different high-temperature treatments. (**A**) 200 °C, OC. (**B**) 400 °C, OC. (**C**) 600 °C, OC. (**D**) 800 °C, OC. (**E**) 200 °C, NTC3. (**F**) 400 °C, NTC3. (**G**) 600 °C, NTC3. (**H**) 800 °C, NTC3.

Figure 5 shows the apparent characteristics of the concrete specimens after undergoing different temperature treatments. After undergoing different temperature treatments, there is a clear difference between the apparent characteristics of ordinary concrete and NTC3 specimens. The volume expansion of both was not apparent between 200 °C and 600 °C, and the surface locally changed from gray to brown. From 800 °C, the surface of the specimens started to show black spots and volume expansion.

### 3.2. Mechanical Properties of Concrete after High Temperature

**Figure 6:**
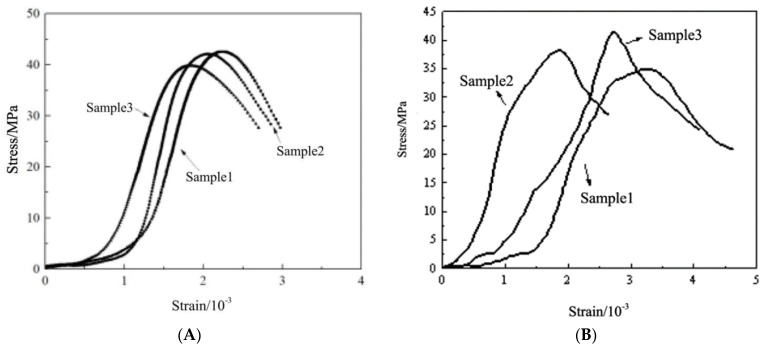

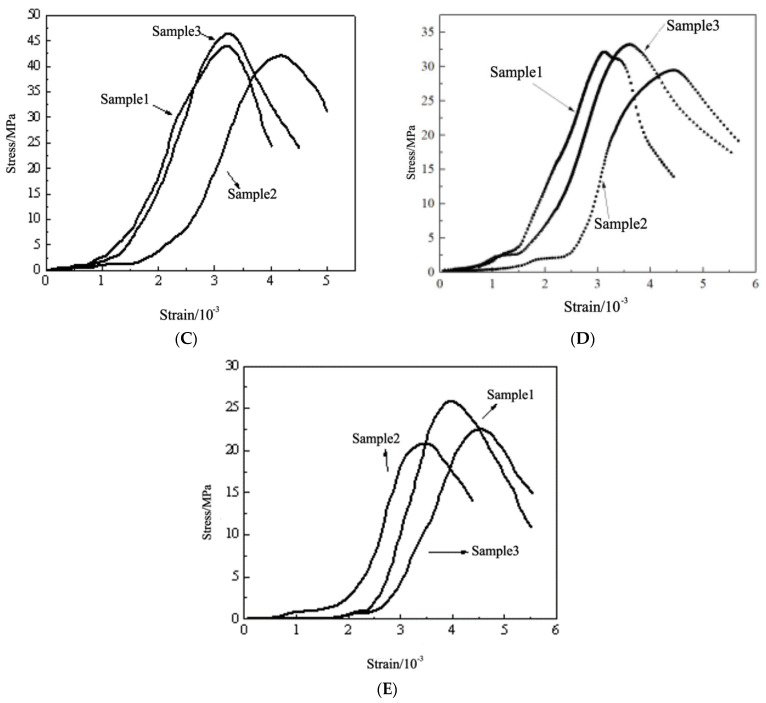
Compressive stress–strain curve of ordinary concrete after high temperature. (**A**) 20 °C. (**B**) 200 °C. (**C**) 400 °C. (**D**) 600 °C. (**E**) 800 °C.

Figure 6 shows the compressive stress–strain curve of ordinary concrete after high temperatures. It can be seen that the stress–strain curve of ordinary concrete at room temperature is divided into four stages: the compacting stage, elastic stage, strengthening stage, and unloading stage. After high-temperature treatment, the stress–strain trend of the specimen has a certain change. After 200 °C, the ordinary concrete test specimen reached the stress peak and did not immediately enter the unloading stage, and the residual strain further expanded. As the treatment temperature was elevated, the average peak compressive strength of ordinary concrete exhibited a further decline. Notably, the compressive strength of ordinary concrete experienced a rapid reduction between 400 °C and 800 °C. At 800 °C, the average peak compressive strength of conventional concrete was measured at 23.12 MPa, indicating a decrease of 44.32% in comparison to the initial measurement.

In this paper, the slope between the peak stress and 0.1 times the peak stress of the test piece is approximately close to the secant modulus. The approximate secant modulus of ordinary concrete is shown in Table 4. It can be seen that the approximate secant modulus of ordinary concrete is 34.646 GPa. After 200 °C high-temperature treatment, the approximate secant modulus of ordinary concrete is reduced to 20.248 GPa. After 400 °C high-temperature treatment, the approximate secant modulus of ordinary concrete is reduced to 16.7 GPa. As the temperature rises further, the approximate secant modulus of ordinary concrete is further reduced. However, the reduction of 400 °C to 800 °C is not significant, and after 800 °C high-temperature treatment, the approximate secant modulus of ordinary concrete is reduced to 13.072 GPa.

### 3.3. Mechanical Properties of NTC3 after High Temperature

**Figure 7:**
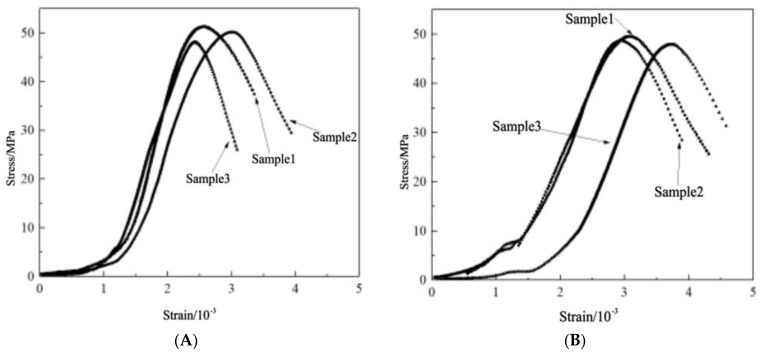

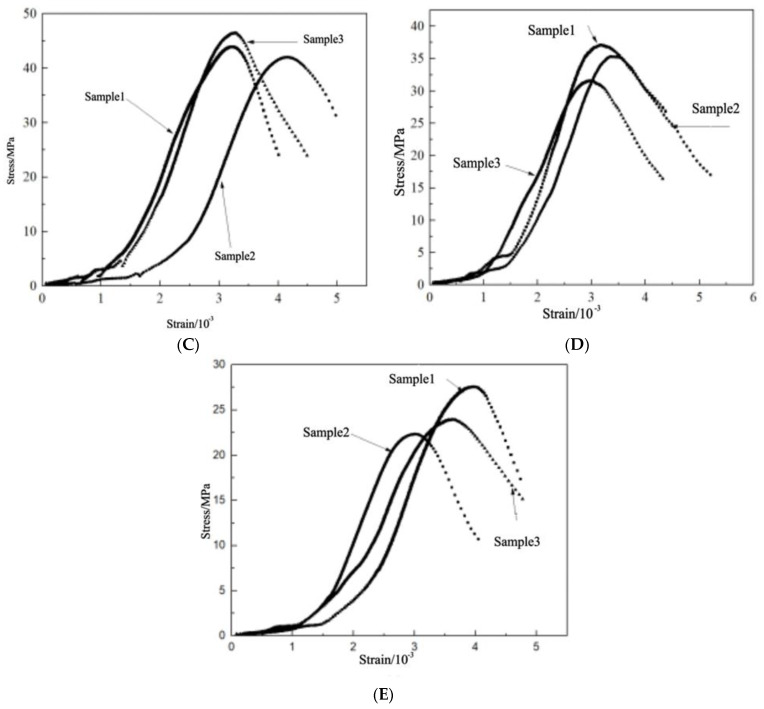
Compressive stress–strain curve of NTC3 after high temperature (**A**) 20 °C. (**B**) 200 °C. (**C**) 400 °C. (**D**) 600 °C. (**E**) 800 °C.

Figure 7 shows the compressive stress–strain curve of NTC3 after high temperature. It can be seen that after high-temperature treatment, the stress–strain trend of the specimen does not change and still presents four stages. At room temperature, the average peak stress of NTC3 was 49.91 MPa. With the further increase of the treatment temperature, the average peak compressive strength of NTC3 is further reduced, similar to ordinary concrete. After 800 °C, the average peak compressive strength of NTC3 is 24.61 MPa, which is 50.69% lower than the initial time. It can be seen that the incorporation of nano-titanium dioxide has an improved effect on the mechanical properties of concrete at temperatures below 600 °C, and it is not helpful to the high-temperature resistance of concrete above 600 °C.

Table 5 displays the estimated secant modulus of the specimen based on the calculation method outlined in the preceding section. The approximate secant modulus of nano-titanium dioxide is 31.229 GPa, and after high-temperature treatment at 200 °C, the approximate secant modulus of the specimen is reduced to 22.173 GPa. After high-temperature treatment at 400 °C, the approximate secant modulus of concrete was reduced to 19.46 GPa. As the temperature rises further, the approximate secant modulus of modified concrete further decreases. After high-temperature treatment at 800 °C, the approximate secant modulus of the specimen was reduced to 10.901 GPa.

## 4. Establishment and Verification of Concrete Damage Constitutive Model

### 4.1. Derivation of the Damage Constitutive Equation

Lamaitre [26] proposed the important strain equivalence principle in 1971: in the uniaxial stress state, it is believed that the constitutive equation of the material can derive the constitutive relationship of the damaged material in the absence of damage as long as the stress in it is replaced by the effective stress. Let the total volume of the specimen be *V*, and the damaged material unit be regarded as various isotropic units. The total volume is composed of two parts, namely the damage zone *V_d_* and the undamaged area *V_n_*, obtaining:

(1) $$\begin{array}{c} V=V_{d}+V_{n} \end{array}$$

Introducing the damage variable *d_c_*, we obtain:

(2) $$\begin{array}{c} d_{c}=\frac{V_{d}}{V} \end{array}$$

Thus, the effective stress *σ_n_* and *σ* can be expressed as

(3) $$\begin{array}{c} \sigma_{n}=\frac{\sigma }{1-d_{c}} \end{array}$$

(4) $$\begin{array}{c} \sigma_{n}=E_{T}\varepsilon \end{array}$$

(5) $$\begin{array}{c} \sigma =E_{T}\left(1-d_{c}\right)\varepsilon \end{array}$$

where *E_T_* is the initial modulus of elasticity after different temperature treatments, and *ε* is the strain [27].

Tensile and compressive strains mainly induce microcrack extension and elongation in near-brittle materials; therefore, the damage evolution path can be described in strain space. According to plasticity theory, the direction of strain in a material depends on the stress state of the material and not on the direction of the incremental stress in the material. Therefore, the multi-axial stress state can be converted into an equivalent stress state by combining the stress invariants, i.e., the equivalent strain *ε_eq_* is

(6) $$\begin{array}{c} \varepsilon_{eq}=\frac{1}{mE_{t}\left(1-\alpha \right)}\left(\alpha {Ι}_{1\sigma }+\sqrt{3J_{2\sigma }}+\beta {\langle \sigma_{ei}^{max}\rangle }^{+}\right) \end{array}$$

where ${Ι}_{1\sigma }$ is the first invariant of the effective stress tensor; $J_{2\sigma }$ is the second invariant of the effective stress tensor; $\sigma_{ei}^{max}$ is the maximum effective principal stress; $\langle \cdot \rangle$ is Macaulay-bracket, which can be expressed as ${\langle x\rangle }^{+}=(x+\left|x\right|)$/2 and ${\langle x\rangle }^{+}=(x-\left|x\right|)/2$.

where

(7) $$\begin{array}{c} {Ι}_{1\sigma }=tr\sigma_{e} \end{array}$$

(8) $$\begin{array}{c} J_{2\sigma }=\frac{1}{2}\sigma_{e}:\sigma_{e}-\frac{1}{6}{Ι}_{1\sigma }^{2} \end{array}$$

(9) $$\begin{array}{c} \sigma_{e}=C_{0}:\varepsilon \end{array}$$

where $\sigma_{e}$ is the second-order effective stress tensor; *tr*(.) is a tensor trace operator; $C_{0}$ is the fourth-order elastic stiffness tensor of undamaged concrete ($A:B=A_{ij}B_{ij})$.

First, define the uniform damage loading surface under strain space as:

(10) $$\begin{array}{c} F\left(\varepsilon_{eq},\kappa \right)=\varepsilon_{eq}-\kappa \leq 0 \end{array}$$

where *κ* is the damage threshold, which represents the cumulative irreversible damage, i.e., the maximum value of the corresponding local equivalent strain throughout the loading process, and defines it as:

(11) $$\begin{array}{c} \kappa \left(t\right)=\underset{r\leq t}{\max}\left(\varepsilon_{eq}\left(t\right)\right) \end{array}$$

Secondly, when the equivalent strain reaches the damage threshold, the concrete begins to suffer, and the initial damage loading surface under the strain space is the ultimate strength surface, which is expressed as:

(12) $$\begin{array}{c} F_{0}=\varepsilon_{eq}-\kappa_{0} \end{array}$$

where *κ*_0_ is the initial damage threshold for concrete, defined as:

(13) $$\begin{array}{c} \kappa_{0}=\frac{f_{c}}{E_{0}} \end{array}$$

Finally, the initial damage loading surface under the strain space after different temperature treatments is written as:

(14) $$\begin{array}{c} F_{t}=\varepsilon_{eq}-\kappa_{t} \end{array}$$

where *κ_t_* is the initial damage threshold after different temperature treatments, defined as:

(15) $$\begin{array}{c} \kappa_{t}=\frac{f_{c}}{E_{T}} \end{array}$$

(16) $$\begin{array}{c} \kappa_{ctb}=\frac{f_{ctb}}{E_{T}} \end{array}$$

where *f_c_* is the peak strength of concrete when uniaxial compression; *f_ctb_* is the stress corresponding to the inflection point before the stress peak; *κ_ctb_* is the damage threshold corresponding to the first inflection point on the stress–strain curve. In the above equation, the strain *ε* and the damage threshold *κ* should be satisfied:

(17) $$\begin{array}{c} \varepsilon =\kappa \end{array}$$

(18) $$\begin{array}{c} \varepsilon_{ctb}=\gamma_{1}\kappa_{ctb} \end{array}$$

(19) $$\begin{array}{c} \varepsilon_{t}=\gamma_{2}\kappa_{ct} \end{array}$$

**Figure 8:**
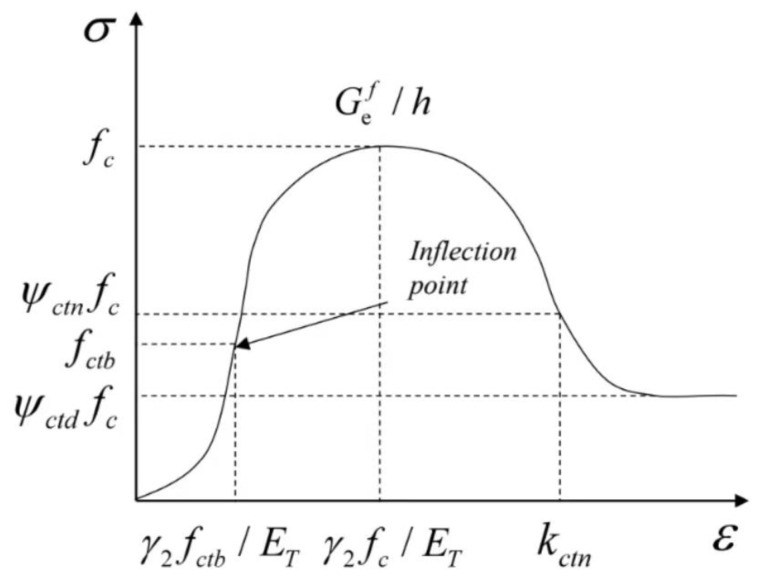
Constitutive relation schematic diagram of uniaxial compression.

The damage evolution equation in line with the actual situation was established through the stress–strain curve obtained by the uniaxial compression test of concrete in this paper. Assuming that $\sigma =f\left(\varepsilon \right)$ is the constitutive relationship under uniaxial compression of concrete and the stress–strain curve should satisfy: when ε=0, σ=0, that is, the curve passes through the coordinate origin. There is only one point on the curve, satisfying dσ/dε=0, that is, there is only one peak point on the curve, at this time $\varepsilon =\varepsilon_{t}$, $\sigma_{t}=f(\varepsilon_{t})$. When $0<\varepsilon <\varepsilon_{ctb}$, $d^{2}\sigma /d\varepsilon^{2}>0$, that is, when the stress just begins to increase, the compacting stage of the concrete test block and the slope of the curve increases with the increase of strain. When $\varepsilon =\varepsilon_{ctb}$, the curve satisfies $\;d^{2}\sigma /d\varepsilon^{2}=0$, which means this point is the first inflection point on the curve, and it is the turning point of the concrete test block from the elastic stress stage to the elastic-plastic stress stage. When $\varepsilon_{ctb}<\varepsilon <\varepsilon_{t}$, $d^{2}\sigma /d\varepsilon^{2}<0$, before the stress reaches the peak point, the slope of the curve decreases with the increase of strain, and there is no inflection point on this curve. When $\varepsilon >\varepsilon_{t}$, there is only one point on the curve that satisfies $d^{2}\sigma /d\varepsilon^{2}=0$, which means this curve has only one inflection point. The strain point where the concrete test block is destroyed, and the bearing capacity is lost. Figure 8 depicts the schematic diagram of the constitutive relationship of concrete under uniaxial compression.

According to the above segmentation conditions for the stress–strain curve of concrete, the stress–strain expression under compression is established. Among them, the ascending section of the curve is expressed exponentially and rationally, and the descending segment of the curve is expressed in quadratic polynomial and exponential forms. Combined with the constitutive relationship (4), the evolution equation of compression damage *d_c_* is derived:

(20) $$\begin{array}{c} d_{c}=\left\{\begin{array}{c} \;\;1-\frac{1}{\gamma_{1}}\times exp\left(\frac{k-\gamma_{1}k_{ctb}}{\gamma_{2}k_{t}/\gamma_{1}}\right)\;\;\;\;\;\;\;\;\;\;\;\;\;\;\;\;\;\;\;\;\;\;\;\;\;\;\;\;\;\;\;\;\;\;\;\;\;\;\;\;\;\;\;\;\;\;\;\;\;\;\;\;\;\;\;\;\;\;\;\;\;\;\;\;\;\;\;\;\;\;\;\;\;\;\;\;\;\;\;0\leq \kappa \leq \kappa_{ctb}\\ 1-\frac{1}{\gamma_{1}{\left(\frac{{\gamma_{2}\kappa }_{t}-\kappa }{\gamma_{2}\kappa_{t}-\gamma_{1}\kappa_{ctb}}\right)}^{\frac{\gamma_{2}}{\gamma_{1}-1}}+\gamma_{2}{\left(\frac{\kappa -\gamma_{1}\kappa_{ctb}}{{\gamma_{2}\kappa }_{t-\gamma_{1}\varepsilon_{ctb}}}\right)}^{\frac{\gamma_{1}}{\gamma_{2}-1}}}\;\;\;\;\;\;\;\;\;\;\;\;\;\;\;\;\;\;\;\;\;\;\;\;\;\;\;\;\;\;\;\;\;\;\;\;\;\;\;\;\;\;\;\;\;\;\;\kappa_{ctb}<\kappa \leq \kappa_{t}\;\;\;\;\\ 1-\frac{\kappa_{t}}{\gamma_{2}\kappa }\left[1-\left(1-\Psi_{ctn}\right){\left(\frac{\kappa -\gamma_{2}\kappa_{t}}{\kappa_{ctn}-{\gamma_{2}\kappa }_{t}}\right)}^{2}\right]\;\;\;\;\;\;\;\;\;\;\;\;\;\;\;\;\;\;\;\;\;\;\;\;\;\;\;\;\;\;\;\;\;\;\;\;\;\;\;\;\;\;\;\;\;\;\;\;\;\;\kappa_{t}<\kappa \leq \kappa_{ctn}\\ \;1-\frac{\kappa_{t}}{\gamma_{2}\kappa }\left[\Psi_{ctd}+\left(\Psi_{ctn}-\Psi_{ctd}\right)\times exp\left(-5\frac{\left(1-\Psi_{ctn}\right)\left(\kappa -\kappa_{ctn}\right)}{\left(\Psi_{ctn}-\Psi_{ctd}\right)\left(\kappa_{ctn}-\kappa_{t}\right)}\right)\right]\;\;\;\;\;\;\;\;\;\;\;\;\;\;\kappa_{ctn}<\kappa \;\; \end{array}\right. \end{array}$$

where, $\gamma_{1}$ is the material constant controlling the compaction stage of concrete, which is taken according to the characteristics of the compression-density phase of the stress–strain curve obtained from the uniaxial compression test of concrete in this paper, and the range of values is defined as 2.5–6.0. $\gamma_{2}$ is the material constant controlling the peak strain of concrete, and the range of values is usually 2.0–4.5. Because high-temperature treatment changes the mechanical properties of the material, concrete treated at different temperatures should correspond to different $\gamma_{1}$ and $\gamma_{2}$. $\Psi_{ctn}$ and $\Psi_{ctd}$ are shape parameters that determine the stress–strain curve, both of which take values in the ranges 0.5–0.9 and 0–0.5, respectively. Where $\kappa_{ctb}$ and $\kappa_{ctn}$ are the inflection point damage thresholds before and after reaching the peak stress point, respectively, indicating the strain value corresponding to the inflection point of the compressive stress–strain curve at the softening section.

### 4.2. Damage Constitutive Model of Ordinary Concrete after High Temperature

**Figure 9:**
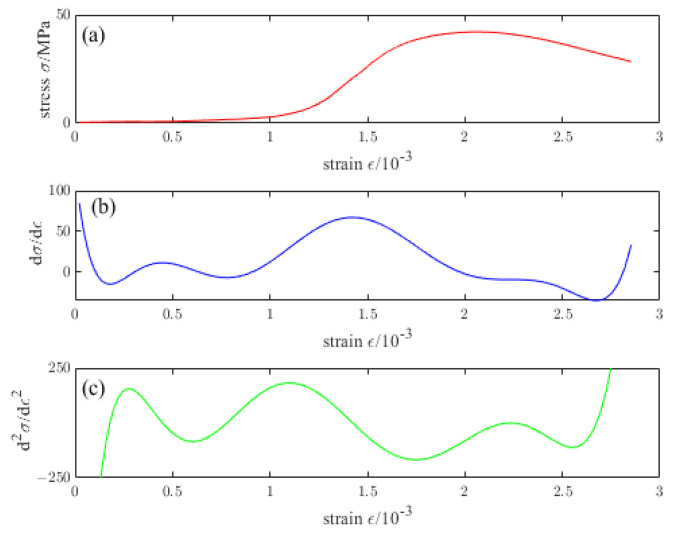
(**a**) Stress–strain curve of ordinary concrete at 20 °C (**b**) First derivative of the stress–strain curve of ordinary concrete at 20 °C (**c**) Second derivative of the stress–strain curve of ordinary concrete at 20 °C.

**Figure 10:**
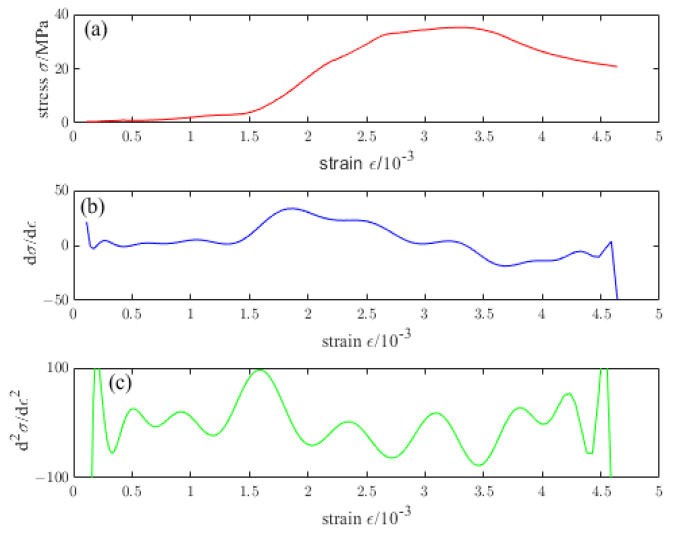
(**a**) Stress–strain curve of ordinary concrete at 200 °C (**b**) First derivative of the stress–strain curve of ordinary concrete at 200 °C (**c**) Second derivative of the stress–strain curve of ordinary concrete at 200 °C.

**Figure 11:**
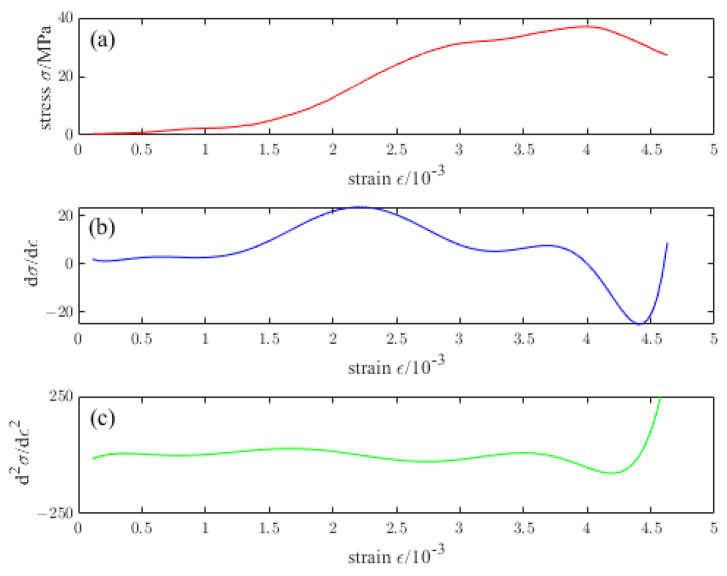
(**a**) Stress–strain curve of ordinary concrete at 400 °C (**b**) First derivative of the stress–strain curve of ordinary concrete at 400 °C (**c**) Second derivative of the stress–strain curve of ordinary concrete at 400 °C.

**Figure 12:**
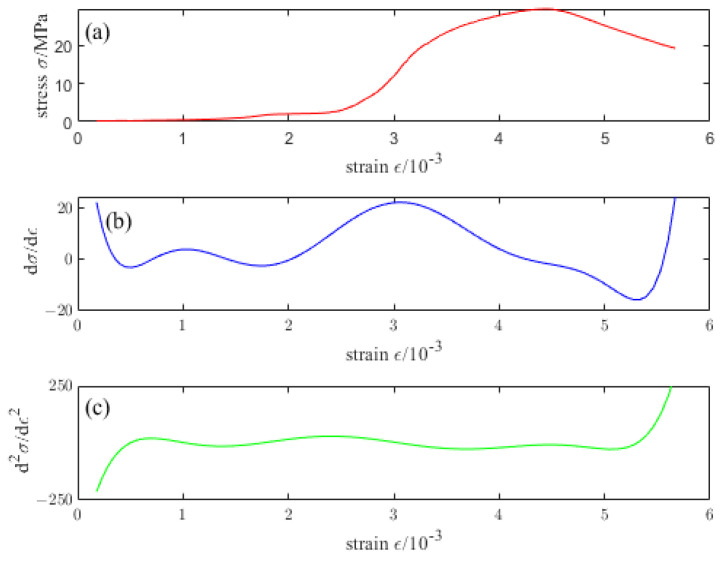
(**a**) Stress–strain curve of ordinary concrete at 600 °C (**b**) First derivative of the stress–strain curve of ordinary concrete at 600 °C (**c**) Second derivative of the stress–strain curve of ordinary concrete at 600 °C.

**Figure 13:**
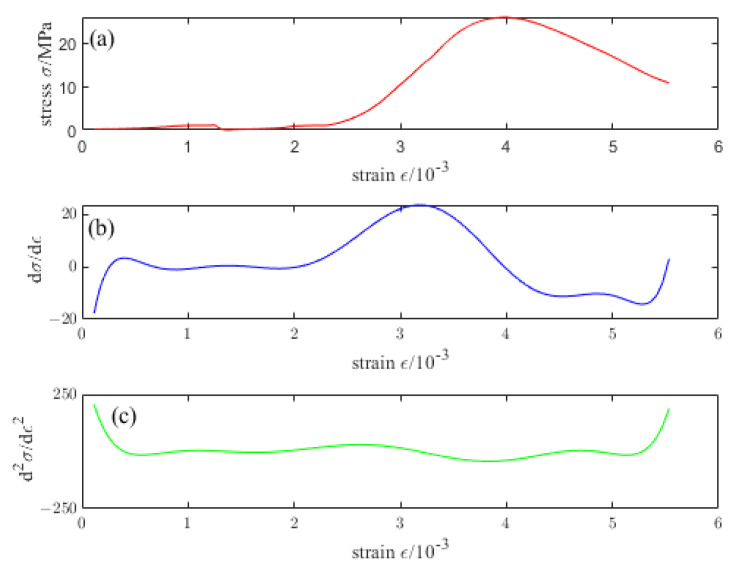
(**a**) Stress–strain curve of ordinary concrete at 800 °C (**b**) First derivative of the stress–strain curve of ordinary concrete at 800 °C (**c**) Second derivative of the stress–strain curve of ordinary concrete at 800 °C.

Figure 9, Figure 10, Figure 11, Figure 12 and Figure 13 are the stress–strain curves of ordinary concrete after different temperature treatments with the first and second derivatives diagrams of the stress–strain curve. Firstly, the stress–strain curve can obtain the peak strain *f_c_* of ordinary concrete after different temperature treatments. Secondly, the initial modulus of elasticity *E_T_* after different temperature treatments can be obtained from the stress–strain curve and the first derivative curve of the stress–strain curve. *κ_t_* can be obtained by substituting *E_T_* into the Equation (15). Finally, the inflection point *κ_ctn_* of the stress–strain curve can be obtained from the first derivative curve of the stress–strain curve. The boundary parameters of concrete compression damage *d_c_* after different temperature treatments are shown in Table 6 and Table 7.

From Equations (5), (15), and (16), combined with the damage evolution parameters in Table 6 and Table 7, the constitutive equations of ordinary concrete after different temperature treatments can be obtained as follows:

(21) $$\begin{array}{c} \sigma =\left\{\begin{array}{c} \frac{E_{T}}{\gamma_{1}}\times exp\left(\frac{k-\gamma_{1}k_{ctb}}{\gamma_{2}k_{t}/\gamma_{1}}\right)\varepsilon \;\;\;\;\;\;\;\;\;\;\;\;\;\;\;\;\;\;\;\;\;\;\;\;\;\;\;\;\;\;\;\;\;\;\;\;\;\;\;\;\;\;\;\;\;\;\;\;\;\;\;\;\;\;\;\;\;\;\;\;\;\;\;\;\;\;\;\;\;\;\;\;\;\;\;\;\;\;\;\;\;\;\;\;0\leq \kappa \leq \kappa_{ctb}\\ \;\;\frac{E_{T}}{\gamma_{1}{\left(\frac{{\gamma_{2}\kappa }_{t}-\kappa }{\gamma_{2}\kappa_{t}-\gamma_{1}\kappa_{ctb}}\right)}^{\frac{\gamma_{2}}{\gamma_{1}-1}}+\gamma_{2}{\left(\frac{\kappa -\gamma_{1}\kappa_{ctb}}{{\gamma_{2}\kappa }_{t-\gamma_{1}\varepsilon_{ctb}}}\right)}^{\frac{\gamma_{1}}{\gamma_{2}-1}}}\varepsilon \;\;\;\;\;\;\;\;\;\;\;\;\;\;\;\;\;\;\;\;\;\;\;\;\;\;\;\;\;\;\;\;\;\;\;\;\;\;\;\;\;\;\;\;\;\;\;\;\;\;\;\;\;\;\kappa_{ctb}<\kappa \leq \kappa_{t}\;\;\;\;\\ \frac{\kappa_{t}E_{T}}{\gamma \kappa }\left[1-\left(1-\Psi_{ctn}\right)\left(\frac{\kappa -\kappa_{t}}{\kappa_{ctn}-\kappa_{t}}\right)\right]\varepsilon \;\;\;\;\;\;\;\;\;\;\;\;\;\;\;\;\;\;\;\;\;\;\;\;\;\;\;\;\;\;\;\;\;\;\;\;\;\;\;\;\;\;\;\;\;\;\;\;\;\;\;\;\;\kappa_{t}<\kappa \leq \kappa_{ctn}\\ \;\frac{\kappa_{t}E_{T}}{\gamma \kappa }\left[\Psi_{ctd}+\left(\Psi_{ctn}-\Psi_{ctd}\right)\times exp\left(-5\frac{\left(1-\Psi_{ctn}\right)\left(\kappa -\kappa_{ctn}\right)}{\left(\Psi_{ctn}-\Psi_{ctd}\right)\left(\kappa_{ctn}-\kappa_{0}\right)}\right)\right]\varepsilon \;\;\;\;\;\;\;\;\;\;\;\kappa_{ctn}<\kappa \;\; \end{array}\right. \end{array}$$

According to Equation (11) and considering the relationship between $\;\varepsilon_{ctb}=\gamma_{1}\kappa_{ctb}$, $\varepsilon_{t}=\gamma_{2}\kappa_{t}$, $\varepsilon_{ctn}=\kappa_{ctn}$, so that Equation (21) can be written as:

(22) $$\begin{array}{c} \sigma =\left\{\begin{array}{c} \frac{E_{T}}{\gamma_{1}}\times exp\left(\frac{\varepsilon -\varepsilon_{ctb}}{\varepsilon_{t}/\gamma_{1}}\right)\varepsilon \;\;\;\;\;\;\;\;\;\;\;\;\;\;\;\;\;\;\;\;\;\;\;\;\;\;\;\;\;\;\;\;\;\;\;\;\;\;\;\;\;\;\;\;\;\;\;\;\;\;\;\;\;\;\;\;\;\;\;\;\;\;\;\;\;\;\;\;\;\;\;\;\;\;\;\;\;\;\;\;\;\;\;\;0\leq \varepsilon \leq \varepsilon_{ctb}\\ \;\;\frac{E_{T}}{\gamma_{1}{\left(\frac{\varepsilon_{t}-\varepsilon }{\varepsilon_{t-\varepsilon_{ctb}}}\right)}^{\frac{\gamma_{2}}{\gamma_{1}-1}}+\gamma_{2}{\left(\frac{\varepsilon -\varepsilon_{ctb}}{\varepsilon_{t-\varepsilon_{ctb}}}\right)}^{\frac{\gamma_{1}}{\gamma_{2}-1}}}\varepsilon \;\;\;\;\;\;\;\;\;\;\;\;\;\;\;\;\;\;\;\;\;\;\;\;\;\;\;\;\;\;\;\;\;\;\;\;\;\;\;\;\;\;\;\;\;\;\;\;\;\;\;\;\;\;\;\;\;\;\;\;\;\;\varepsilon_{ctb}<\varepsilon \leq \varepsilon_{t}\;\;\;\;\\ \frac{\varepsilon_{t}E_{T}}{\gamma_{2}}\left[1-\left(1-\Psi_{ctn}\right){\left(\frac{\varepsilon -\varepsilon_{t}}{\varepsilon_{ctn}-\varepsilon_{t}}\right)}^{2}\right]\;\;\;\;\;\;\;\;\;\;\;\;\;\;\;\;\;\;\;\;\;\;\;\;\;\;\;\;\;\;\;\;\;\;\;\;\;\;\;\;\;\;\;\;\;\;\;\;\;\;\;\;\;\;\;\;\;\;\varepsilon_{t}<\varepsilon \leq \varepsilon_{ctn}\\ \frac{\varepsilon_{t}E_{T}}{\gamma_{2}}\left[\Psi_{ctd}+\left(\Psi_{ctn}-\Psi_{ctd}\right)\times exp\left(-5\frac{\left(1-\Psi_{ctn}\right)\left(\varepsilon -\varepsilon_{ctn}\right)}{\left(\Psi_{ctn}-\Psi_{ctd}\right)\left(\varepsilon_{ctn}-\frac{\varepsilon_{t}}{\gamma_{2}}\right)}\right)\right]\;\;\;\;\;\;\;\;\;\;\;\;\;\;\;\;\varepsilon_{ctn}<\varepsilon \;\; \end{array}\right. \end{array}$$

**Figure 14:**
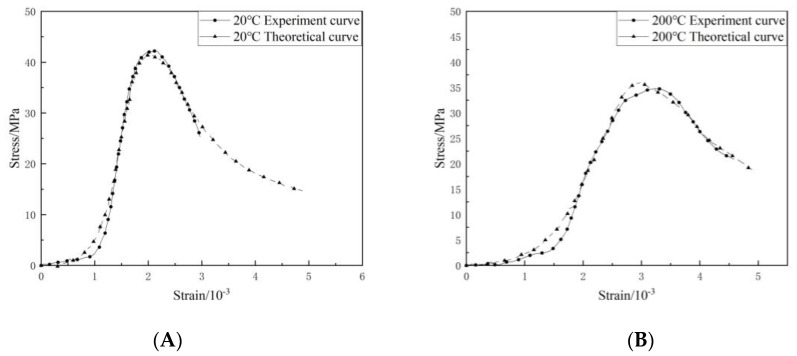

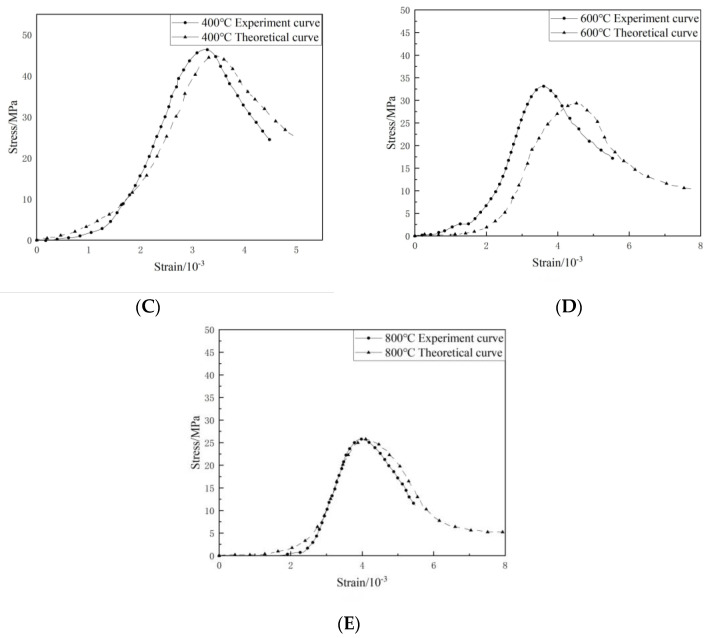
Comparison of constitutive equation curve and test curve of ordinary concrete at different temperatures. (**A**) 20 °C. (**B**) 200 °C. (**C**) 400 °C. (**D**) 600 °C. (**E**) 800 °C.

Figure 14 compares the theoretical and test curves of the constitutive equation of ordinary concrete after different temperature treatments. It can be seen from the figure that the theoretical curve of ordinary concrete constitutive equation is in good agreement with the test curve, indicating that the damage constitutive model established in this paper can better reflect the stress–strain curve characteristics of concrete uniaxial compression, which has certain practical engineering significance.

### 4.3. Damage Constitutive Model of NTC3 after High Temperature

The test curve and the theoretical curve of the constitutive equation for ordinary concrete established in the preceding section correspond very well. The stress–strain test curve for uniaxial compression of nano-modified concrete demonstrates that the trend of the stress–strain curve is consistent with that of ordinary concrete. The peak strength and high-temperature resistance of concrete were improved after adding nanometers. When the temperature ranges from 20 °C to 600 °C, the peak strength of NTC3 increases by 20%. In comparison, when the temperature exceeds 600 °C, the enhancement of the peak strength of the specimen by the addition of TiO_2_ nanoparticles is not significant. The strengthening coefficient can be defined to reflect the strength increase effect of nano-titanium dioxide on concrete. The strengthening coefficient can be obtained by fitting the peak strength change value of concrete before and after nano addition. The strengthening coefficient is defined as:

(23) $$Q_{f}=\frac{f_{c}^{n}}{f_{c}}$$

where *Q_f_* reflects the strengthening coefficient brought by the addition of nanomaterials to concrete under different temperatures. $f_{c}^{n}$ is the uniaxial compressive strength of NTC3 under different temperatures. *f_c_* is the uniaxial compressive strength of ordinary concrete under different temperatures.

**Figure 15:**
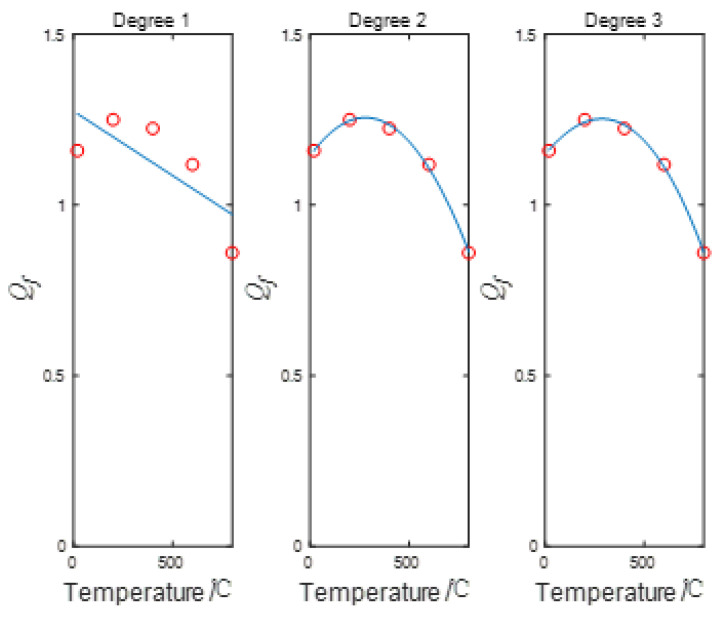
The strengthening coefficient *Q_f_* is about the primary, secondary, and cubic polynomial fitting relationship of the processing temperature.

Table 8 shows the strengthening coefficient *Q_f_* of the peak strength of NTC3 compared with ordinary concrete after different temperature treatments. Figure 15 shows the primary, secondary, and cubic polynomial fitting of the strengthening coefficient *Q_f_* with respect to the processing temperature. The fitting degree of the quadratic polynomial is good, and the goodness of fit (*R*^2^) of the curve is 0.999. Therefore, this relationship can be used to predict the uniaxial compressive strength of NTC3 at different temperatures. The fitting relation is:

(24) $$\begin{array}{c} Q_{f}=-1.445\times 10^{-6}T^{2}+8.0630\times 10^{-4}T+1.1431 \end{array}$$

Because the uniaxial compressive stress–strain curve of concrete after adding TiO_2_ nanopowder is consistent with the stress–strain curve trend of ordinary concrete, it only improves at the peak point. Therefore, the strengthening coefficient can be introduced on the basis of the second and third stages of the ordinary concrete constitutive equation established above, and the damage constitutive equation of NTC3 under different temperatures can be obtained. The damage constitutive model of NTC3 after high temperature and the derivation process of the damage evolution equation have been described in the previous section and will not be described here.

**Figure 16:**
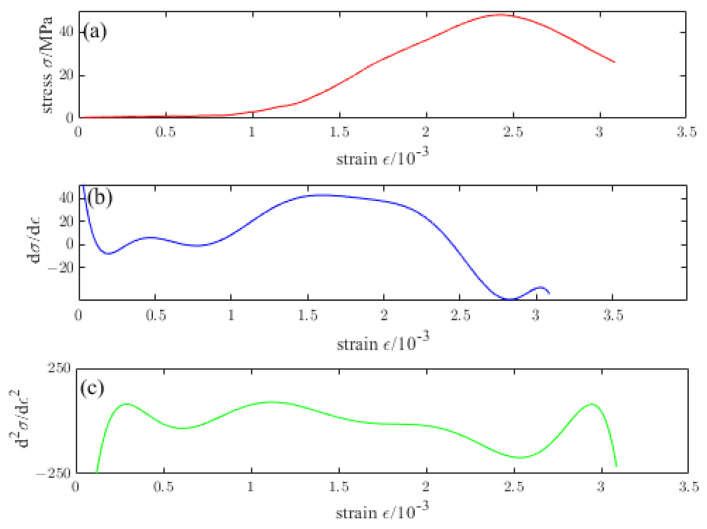
(**a**) Stress–strain curve of NTC3 at 20 °C (**b**) First derivative of the stress–strain curve of NTC3 at 20 °C (**c**) Second derivative of the stress–strain curve of NTC3 at 20 °C.

**Figure 17:**
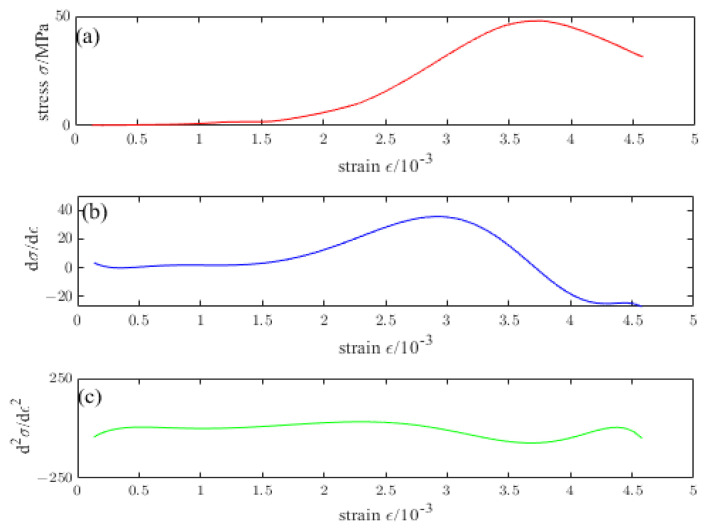
(**a**) Stress–strain curve of NTC3 at 200 °C (**b**) First derivative of the stress–strain curve of NTC3 at 200 °C (**c**) Second derivative of the stress–strain curve of NTC3 at 200 °C.

**Figure 18:**
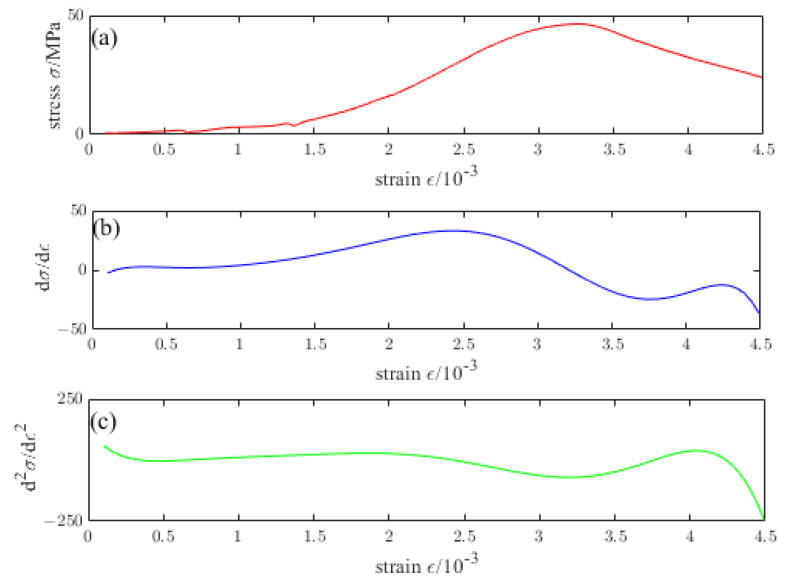
(**a**) Stress–strain curve of NTC3 at 400 °C (**b**) First derivative of the stress–strain curve of NTC3 at 400 °C (**c**) Second derivative of the stress–strain curve of NTC3 at 400 °C.

**Figure 19:**
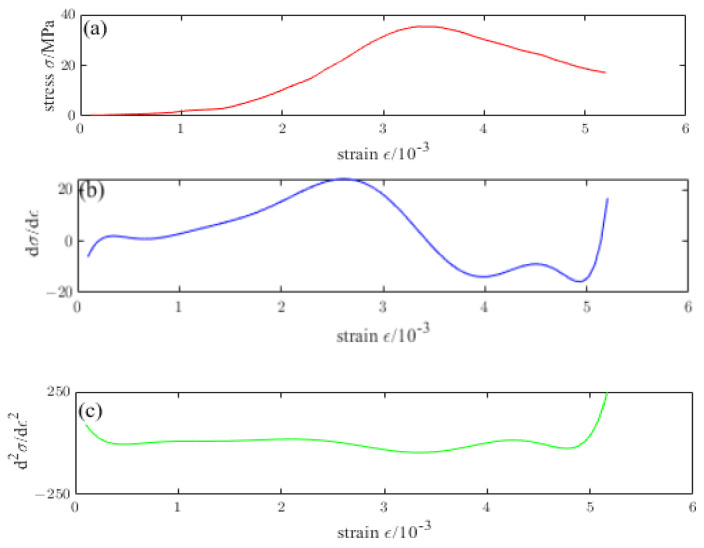
(**a**) Stress–strain curve of NTC3 at 600 °C (**b**) First derivative of the stress–strain curve of NTC3 at 600 °C (**c**) Second derivative of the stress–strain curve of NTC3 at 600 °C.

**Figure 20:**
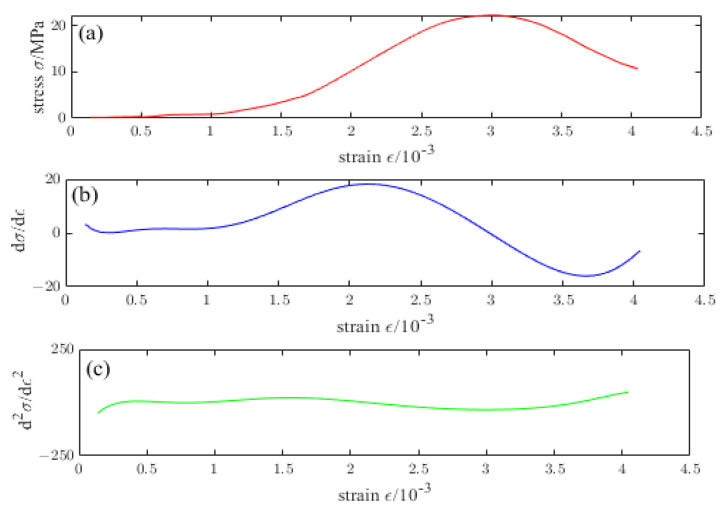
(**a**) Stress–strain curve of NTC3 at 800 °C (**b**) First derivative of the stress–strain curve of NTC3 at 800 °C (**c**) Second derivative of the stress–strain curve of NTC3 at 800 °C.

Figure 16, Figure 17, Figure 18, Figure 19 and Figure 20 are the first and second derivatives of the stress–strain curve and stress–strain curve of NTC3 after different temperature treatments. Firstly, the peak strain $f_{c}^{n}$ of NTC3 after different temperature treatments can be obtained from the stress–strain curve. Secondly, the initial modulus of elasticity can be *E*_T_ obtained from the stress–strain curve and the first derivative curve of the stress–strain curve. The *κ_t_* can be obtained by substituting it into Equation (15). Finally, the inflection point *κ_ctn_* of the stress–strain curve can be obtained from the first derivative curve of the stress–strain curve. The boundary parameters of NTC3 compression damage *d*_c_ after different temperature treatments are shown in Table 9 and Table 10.

First, the Equation (16) can obtain the damage evolution equation of NTC3 under different temperatures, that is

(25) $$\begin{array}{c} \;d_{c}=\left\{\begin{array}{c} 1-\frac{1}{\gamma_{1}}\times exp\left(\frac{k-\gamma_{1}k_{ctb}}{\gamma_{2}k_{t}/\gamma_{1}}\right)\;\;\;\;\;\;\;\;\;\;\;\;\;\;\;\;\;\;\;\;\;\;\;\;\;\;\;\;\;\;\;\;\;\;\;\;\;\;\;\;\;\;\;\;\;\;\;\;\;\;\;\;\;\;\;\;\;\;\;\;\;\;\;\;\;\;\;\;\;\;\;\;\;\;\;\;\;\;\;0\leq \kappa \leq \kappa_{ctb}\\ 1-\frac{1}{\gamma_{1}{\left(\frac{{\gamma_{2}\kappa }_{t}-\kappa }{\gamma_{2}\kappa_{t}-\gamma_{1}\kappa_{ctb}}\right)}^{\frac{\gamma_{2}}{\gamma_{1}-1}}+\gamma_{2}{\left(\frac{\kappa -\gamma_{1}\kappa_{ctb}}{{\gamma_{2}\kappa }_{t-\gamma_{1}\varepsilon_{ctb}}}\right)}^{\frac{\gamma_{1}}{\gamma_{2}-1}}}\;\;\;\;\;\;\;\;\;\;\;\;\;\;\;\;\;\;\;\;\;\;\;\;\;\;\;\;\;\;\;\;\;\;\;\;\;\;\;\;\;\;\;\;\kappa_{ctb}<\kappa \leq \kappa_{t}\;\;\;\;\\ 1-\frac{\kappa_{t}}{\gamma_{2}\kappa }\left[1-\left(1-\Psi_{ctn}\right){\left(\frac{\kappa -\gamma_{2}\kappa_{t}}{\kappa_{ctn}-{\gamma_{2}\kappa }_{t}}\right)}^{2}\right]\;\;\;\;\;\;\;\;\;\;\;\;\;\;\;\;\;\;\;\;\;\;\;\;\;\;\;\;\;\;\;\;\;\;\;\;\;\;\;\;\;\;\;\;\;\;\;\;\;\;\kappa_{t}<\kappa \leq \kappa_{ctn}\\ 1-\frac{\kappa_{t}}{\gamma_{2}\kappa }\left[\Psi_{ctd}+\left(\Psi_{ctn}-\Psi_{ctd}\right)\times exp\left(-5\frac{\left(1-\Psi_{ctn}\right)\left(\kappa -\kappa_{ctn}\right)}{\left(\Psi_{ctn}-\Psi_{ctd}\right)\left(\kappa_{ctn}-\kappa_{t}\right)}\right)\right]\;\;\;\;\;\;\;\;\;\;\;\;\;\;\kappa_{ctn}<\kappa \;\; \end{array}\right. \end{array}$$

Secondly, the damage constitutive equation of NTC3 under different temperatures can be obtained by substituting (25) into Equation (5).

(26) $$\begin{array}{c} \sigma =\left\{\begin{array}{c} \frac{E_{T}}{\gamma_{1}}\times exp\left(\frac{\varepsilon -\varepsilon_{ctb}}{\varepsilon_{t}/\gamma_{1}}\right)\varepsilon \;\;\;\;\;\;\;\;\;\;\;\;\;\;\;\;\;\;\;\;\;\;\;\;\;\;\;\;\;\;\;\;\;\;\;\;\;\;\;\;\;\;\;\;\;\;\;\;\;\;\;\;\;\;\;\;\;\;\;\;\;\;\;\;\;\;\;\;\;\;\;\;\;\;\;\;\;\;\;\;\;\;\;0\leq \varepsilon \leq \varepsilon_{ctb}\\ \;\;\frac{E_{T}}{\gamma_{1}{\left(\frac{\varepsilon_{t}-\varepsilon }{\varepsilon_{t-\varepsilon_{ctb}}}\right)}^{\frac{\gamma_{2}}{\gamma_{1}-1}}+\gamma_{2}{\left(\frac{\varepsilon -\varepsilon_{ctb}}{\varepsilon_{t-\varepsilon_{ctb}}}\right)}^{\frac{\gamma_{1}}{\gamma_{2}-1}}}\varepsilon \;\;\;\;\;\;\;\;\;\;\;\;\;\;\;\;\;\;\;\;\;\;\;\;\;\;\;\;\;\;\;\;\;\;\;\;\;\;\;\;\;\;\;\;\;\;\;\;\;\;\;\;\;\;\;\;\;\;\;\;\varepsilon_{ctb}<\varepsilon \leq \varepsilon_{t}\;\;\;\;\\ \;\frac{\varepsilon_{t}E_{T}}{\gamma_{2}}\left[1-\left(1-\Psi_{ctn}\right){\left(\frac{\varepsilon -\varepsilon_{t}}{\varepsilon_{ctn}-\varepsilon_{t}}\right)}^{2}\right]\;\;\;\;\;\;\;\;\;\;\;\;\;\;\;\;\;\;\;\;\;\;\;\;\;\;\;\;\;\;\;\;\;\;\;\;\;\;\;\;\;\;\;\;\;\;\;\;\;\;\;\;\;\;\;\;\;\;\varepsilon_{t}<\varepsilon \leq \varepsilon_{ctn}\\ \frac{\varepsilon_{t}E_{T}}{\gamma_{2}}\left[\Psi_{ctd}+\left(\Psi_{ctn}-\Psi_{ctd}\right)\times exp\left(-5\frac{\left(1-\Psi_{ctn}\right)\left(\varepsilon -\varepsilon_{ctn}\right)}{\left(\Psi_{ctn}-\Psi_{ctd}\right)\left(\varepsilon_{ctn}-\frac{\varepsilon_{t}}{\gamma_{2}}\right)}\right)\right]\;\;\;\;\;\;\;\;\;\;\;\;\;\;\;\;\varepsilon_{ctn}<\varepsilon \;\; \end{array}\right.\;\; \end{array}$$

**Figure 21:**
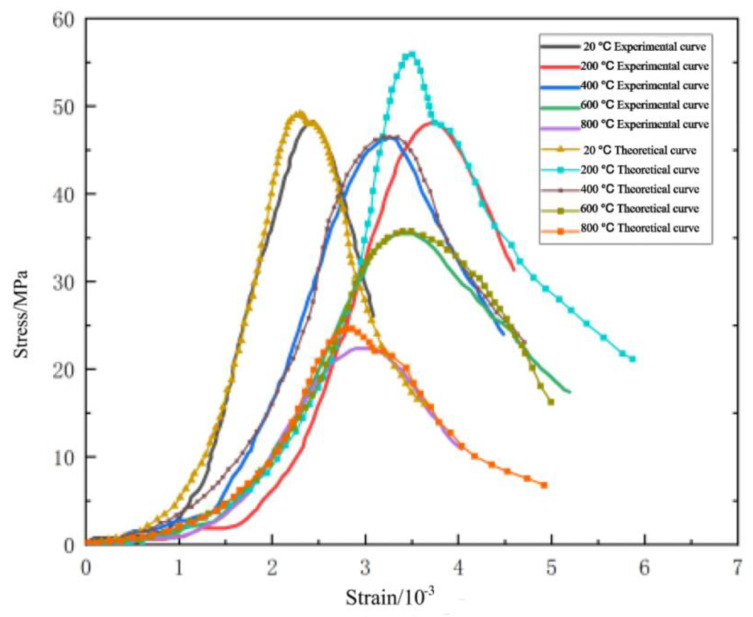
Comparison of NTC3 constitutive equation curve and test curve at different temperatures.

Figure 21 compares the theoretical and test curves of the constitutive equation of NTC3 after different temperature treatments. From the figure, the theoretical curve of NTC3 constitutive equation is in good agreement with the experimental curve, indicating that the NTC3 damage constitutive model established in this paper can better reflect the stress–strain curve characteristics of NTC3 uniaxial compression, which verifies the rationality and reliability of the model and the model parameter determination method, which has certain practical significance.

## 5. Conclusions

In this paper, the mechanical properties of ordinary concrete and NTC3 after high temperature are analyzed and compared, mainly through experimental and theoretical analysis, and the damage constitutive model of ordinary concrete and NTC3 is established.

(1) Ordinary concrete and NTC3 specimens were subjected to various temperatures ranging from 20 °C to 800 °C. Notably, the volume expansion of both materials was not apparent within the temperature range of 200 °C to 600 °C. Additionally, the surface color of the specimens transitioned from gray to brown. At a temperature of 800 °C, the specimen exhibits the formation of black spots on its surface and undergoes volumetric expansion.
(2) With the increase of temperature, ordinary concrete’s average peak compressive resistance is further reduced. At 800 °C, the average peak compressive strength of ordinary concrete was 23.120 MPa, which decreased by 44.32% compared with the initial time, and the approximate secant modulus decreased to 13.072 GPa. Similar to ordinary concrete, after high-temperature treatment from 400 °C to 800 °C, the compressive strength of NTC3 decreased, and the average peak compressive strength of NTC3 was 24.610 MPa at 800 °C, a decrease of 50.69% compared with the initial time. It can be seen that the incorporation of nano titanium dioxide at lower than 600 °C can help the high-temperature resistance of concrete, and when it is higher than 600 °C, the mechanical properties of ordinary concrete and NTC3 compression are basically no different.
(3) The stress–strain curves exhibited by ordinary concrete specimens subjected to uniaxial compression and titanium nanoconcrete specimens prior to and subsequent to high-temperature exposure exhibit similar shapes and characteristics. A constitutive relationship formula was established for the stress–strain curve of ordinary concrete specimens and NTC3 under uniaxial compression. A constitutive relationship for the uniaxial compressive stress–strain behavior of concrete was established, taking into account the variable of high-temperature damage. The constitutive relationship of concrete under high temperatures has been enhanced in accordance with the law of continuous change.

## Figures and Tables

**Table 1:** Chemical composition of PO42.5 Portland cement/%.

Element	SiO_2_	Al_2_O_3_	CaO	Fe_2_O_3_	MgO	SO_3_
Cement	22.02	5.2	64.42	5.23	1.02	2.1

**Table 2:** Physical properties of nano-titanium dioxide.

Element	Density/ g/cm^3^	Melting Point °C	Boiling Point °C	Particle Size nm
nano-titanium dioxide	4.260	1855	2900	25

**Table 3:** Mixture ratios of concrete specimen (OC for ordinary concrete).

Group	Cement (kg/m^3^)	Limestone (kg/m^3^)	Sand (kg/m^3^)	Water (kg/m^3^)	Nano-Titanium Dioxide (kg/m^3^)
OC	415	1224	583	177	0
NTC3	402.550	1224	583	177	12.450

**Table 4:** Influence of different temperatures on the mechanical properties of ordinary concrete.

Temperature/°C	No.	Peak Stress/MPa	Peak Strain/10^−3^	0.1 Times the Peak Stress/MPa	0.1 Times the Peak Strain/10^−3^	Approximate Secant Modulus/GPa
20	1	42.630	2.234	4.263	1.035	31.999
	2	42.130	2.057	4.213	1.087	39.090
	3	39.820	1.835	3.982	0.744	32.849
	average value	41.527	2.042	4.152	0.955	34.646
	standard deviation	1.224	0.163	0.122	1.151	3.161
200	1	35.190	3.304	3.519	1.448	17.064
	2	38.140	1.845	3.814	0.356	23.053
	3	41.690	2.739	4.169	0.920	20.627
	average value	37.943	3.558	3.794	1.448	16.700
	standard deviation	2.344	0.433	0.234	0.233	3.348
400	1	41.170	2.961	4.117	1.199	21.029
	2	35.670	3.741	3.567	1.759	16.197
	3	36.990	3.972	3.699	1.386	12.874
	average value	37.943	3.558	3.794	1.448	16.700
	standard deviation	2.344	0.433	0.234	0.233	3.348
600	1	32.140	3.139	3.214	1.400	16.634
	2	29.450	4.442	2.945	2.514	13.747
	3	33.170	3.616	3.317	1.626	15.002
	standard deviation	1.569	0.538	0.157	0.481	1.182
	average value	31.587	3.732	3.159	1.847	15.128
800	1	25.960	4.007	2.596	2.534	15.862
	2	20.880	3.430	2.088	1.881	12.132
	3	22.520	4.535	2.252	2.729	11.223
	average value	23.120	3.991	2.312	2.381	13.072
	standard deviation	2.117	0.451	0.212	0.363	2.007

**Table 5:** Effect of different temperatures on mechanical properties of nano-carbon dioxide concrete.

Temperature/°C	No.	Peak Stress/MPa	Peak Strain/10^−3^	0.1 Times the Peak Stress/MPa	0.1 Times the Peak Strain/10^−3^	Approximate Secant Modulus/GPa
20	1	51.370	2.568	5.137	1.192	33.600
	2	50.220	3.019	5.022	1.332	26.792
	3	48.130	2.427	4.813	1.126	33.295
	average value	49.907	2.671	4.991	1.217	31.229
	standard deviation	1.341	0.252	0.134	0.859	3.140
200	1	49.60	3.068	4.960	1.460	20.977
	2	48.680	2.940	4.868	0.975	22.296
	3	47.910	3.738	4.791	1.883	23.245
	average value	48.730	3.249	4.873	1.439	22.173
	standard deviation	0.691	0.350	0.070	0.371	0.930
400	1	43.950	3.220	4.395	1.179	19.380
	2	42.010	4.163	4.201	2.014	17.594
	3	46.430	3.284	4.643	1.332	21.407
	average value	44.130	3.556	4.413	1.508	19.46
	standard deviation	1.809	0.430	0.181	0.363	1.558
600	1	37.150	3.170	3.715	1.146	16.519
	2	35.320	3.361	3.532	1.499	17.072
	3	31.520	2.984	3.152	1.120	15.219
	average value	34.663	3.172	3.466	1.255	16.270
	standard deviation	2.345	0.154	0.234	0.173	0.777
800	1	27.580	3.961	2.758	1.775	11.355
	2	22.320	3.012	2.232	1.330	11.943
	3	23.920	3.619	2.392	1.330	9.405
	average value	24.607	3.531	2.461	1.4780	10.901
	standard deviation	2.202	0.392	0.220	0.210	1.085

**Table 6:** Parameters in the ascending section of stress–strain curve after different temperature treatment.

Temperature/°C	$E_{T}$ $/\times 10^{3}MPa$	$f_{ctb}/MPa$	$\gamma_{1}$	$\kappa_{ctb}$ $/\times 10^{-3}$	$\gamma_{1}\kappa_{ctb}$ $/\times 10^{-3}$
20	70.8039	21.307	4.759893	0.300931	1.4324
200	31.4246	16.137	3.876739	0.513524	1.9908
400	22.2528	18.239	2.721436	0.819641	2.2306
600	30.2136	14.205	6.508785	0.470149	3.0601
800	26.7927	14.784	5.805952	0.551796	3.2037

**Table 7:** Parameters in the descending section of stress–strain curve after different temperature treatment.

Temperature/°C	$f_{c}/MPa$	$\gamma_{2}$	$\kappa_{t}$ $/\times 10^{-3}$	$\gamma_{2}\kappa_{t}$ $/\times 10^{-3}$	$\kappa_{ctn}$
20	41.53	3.4	0.586550	1.349064	2.6664
200	35.19	2.8	1.119823	3.355044	3.8865
400	36.96	2.4	1.660915	3.986195	4.5632
600	29.45	4.6	0.974780	4.483986	5.3598
800	25.96	4.1	0.968854	3.993900	5.5234

**Table 8:** The strengthening coefficient *Q_f_* of NTC3 compared with the peak strength of ordinary concrete after different temperature treatment.

Temperature/℃	$f_{c}^{n}$ /MPa	$f_{c}$ /MPa	$Q_{f}$
20	48.13	41.53	1.159
200	47.91	38.34	1.250
400	46.43	37.94	1.224
600	35.32	31.59	1.118
800	22.32	25.96	0.860

**Table 9:** Parameters in the ascending section of NTC3 stress–strain curve after different temperature treatment.

Temperature/°C	$E_{T}$ $/(10^{3}MPa)$	$f_{ctb}$ /(MPa)	$\gamma_{1}$	$\kappa_{ctb}$ $/(10^{-3})$	$\gamma_{1}\kappa_{ctb}$ $/(10^{-3})$
20	46.08	20.410	3.6	0.443	1.596
200	33.50	29.483	3.3	0.880	2.920
400	31.83	29.170	2.7	0.916	2.429
600	23.95	22.703	2.8	0.948	2.615
800	17.47	12.514	3.0	0.716	2.129

**Table 10:** Parameters in the descending section of NTC3 stress–strain curve after different temperature treatment.

Temperature/°C	$f_{c}^{n}/(MPa)$	$\gamma_{2}$	$\kappa_{t}$ $/(10^{-3})$	$\gamma_{2}\kappa_{t}$ $/(10^{-3})$	$\kappa_{ctn}$
20	48.13	2.3	1.044	2.427	2.824
200	47.91	2.6	1.430	3.738	4.292
400	46.43	2.3	1.459	3.284	3.758
600	35.32	2.3	1.475	3.361	4.931
800	22.32	2.4	1.278	3.012	3.665

## Data Availability

Data is contained within the article.
